# Model-theoretic semantics and revenge paradoxes

**DOI:** 10.1007/s11098-018-1041-7

**Published:** 2018-02-02

**Authors:** Lorenzo Rossi

**Affiliations:** Department of Philosophy (KGW), University of Salzburg, Franziskanergasse 1, 5020 Salzburg, Austria

**Keywords:** Model-theoretic semantics, Semantic paradoxes, Revenge paradoxes, Model-theoretic instrumentalism

## Abstract

Revenge arguments purport to show that any proposed solution to the semantic paradoxes generates new paradoxes that prove that solution to be inadequate. In this paper, I focus on revenge arguments that employ the model-theoretic semantics of a target theory and I argue, *contra* the current revenge-theoretic wisdom, that they can constitute genuine expressive limitations. I consider the anti-revenge strategy elaborated by Field (J Philos Log 32:139–177, 2003; Revenge of the Liar, Oxford University Press, Oxford, pp 53–144, 2007; Saving truth from paradox, Oxford University Press, Oxford, 2008, §§21–23) and argue that it does not offer a way out of the revenge problem. More generally, I argue that the difference between ‘standard’ and ‘revenge’ paradoxes is ill-conceived and should be abandoned. This will contribute to show that the theories that provide a uniform account of truth and other semantic notions are the ones best equipped to avoid the paradoxes altogether—‘standard’ and ‘revenge’ alike.

## Introduction

*Prima facie*, revenge paradoxes can be characterized as arguments to the effect that any proposed solution to the semantic paradoxes generates new paradoxes that prove that solution to be inadequate. Such paradoxes often make use of notions employed in the theories they are directed against, and are argued to be similar to the ‘standard’ semantic paradoxes, such as the Liar and Curry’s paradox (see e.g. Field 2007).^1^ Revenge arguments are typically used to conclude that revenge-prone theories do not solve the semantic paradoxes in general: even though they avoid the ‘standard’ semantic paradoxes, they suffer from new, structurally similar antinomies, that can only be avoided at the cost of significant expressive limitations.

A straightforward revenge strategy involves the *model-theoretic* (henceforth ‘MT’) semantics of a theory of truth. MT-revenge arguments point to the inexpressibility in a theory *T* of some notion that is definable in or justified by the MT-semantics for *T*. For instance, let $\lambda$ be a sentence equivalent to ‘$\lambda$ is not true’, and let *T* be a non-classical theory of truth in which $\lambda$ is not in the extension of the truth predicate, nor in the extension of the negated truth predicate.^2^ In the MT-semantics for *T*, it is typically possible to define a predicate for ‘determinateness’ that captures the status of $\lambda$, declaring it to be not determinate. However, expressing such a predicate in *T* makes the theory *Ttrivial*—i.e. it forces *T* to contain every sentence of its language—and hence such a predicate is inexpressible in *T*. The inexpressibility of the model-theoretical notion of ‘determinateness’ provides an example of an MT-revenge paradox. As we will see below, the MT-revenge paradox just sketched can be adapted to essentially all non-classical theories of truth.^3^

The importance of MT-revenge derives from its scope. Most theories of truth come with an MT-semantics, which is used to provide an interpretation for them, or to prove them non-trivial. However, MT-revenge paradoxes are often considered to be as easy to construct as they are to defuse, and ultimately unproblematic.^4^ In this paper, I challenge this view. In order to do so, I consider the vigorous defence against MT-revenge elaborated by Field (2007, 2008, §§21–23). Field’s anti-revenge strategy can be applied to every theory for which an MT-semantics can be given: if successful, it promises to shield every theory of truth from revenge attacks based on MT-semantics. In a nutshell, Field argues that MT-revenge arguments depend on a confusion between *model-independent* and *model-relative* semantic notions: MT-revenge paradoxes employ the latter, but only the former can be used to characterize genuine semantic notions, and hence to give rise to genuine semantic paradoxes. However, I will argue that this distinction does not provide an acceptable ground to distinguish between genuine and non-genuine semantic notions, and thus it does not offer a way out of the revenge problem. More generally, I will argue that the difference between ‘standard’ and ‘revenge’ paradoxes is ill-conceived and should ultimately be abandoned. This will contribute to show that the theories that provide a *uniform* account of truth and other semantic notions are the ones best equipped to avoid the paradoxes altogether.^5^

The purpose of this paper is not to argue that *every* MT-revenge argument is successful. As Beall (2007) has warned, MT-revenge paradoxes can be very simple to formulate, and this can make the resulting arguments ‘too easy’, and ultimately uninteresting. In order to work, revenge arguments must have a proper justification, and their success can only be determined on a case-by-case basis. What I will show is that MT-revenge arguments do not fail *qua* model-theoretic: if they fail, they do so so for specific reasons, such as an unconvincing justification or lack of importance.

The paper is structured as follows. In Sect. 2 I introduce some representative ‘standard’ and ‘revenge’ semantic paradoxes. In Sect. 3 I present Field’s anti-revenge strategy, and in Sect. 4 I argue that it fails to neutralize MT-revenge arguments. In Sect. 5 I discuss a possible rejoinder, namely the idea that the MT-semantics is a mere tool to prove non-triviality results and, as such, it does not characterize genuine semantic notions; I argue that this reply also fails to neutralize MT-revenge arguments. Section 6 concludes.

## Paradoxes and revenge

Semantic paradoxes arise from a combination of three factors: (i) classical logic;^6^ (ii) a *modicum* of syntax; (iii) *naïveté* about truth, namely the idea that for every sentence $\varphi$, $\varphi$ and ‘$\varphi$ is true’ are (in some sense) equivalent. To see this, consider a first-order language ${\mathcal {L}}_{\mathsf{Tr}}$ that contains the predicate ${\mathsf {True}}$ (for ‘$\ldots$ is true’), and a theory *T* formulated in ${\mathcal {L}}_\mathsf{Tr}$ that obeys classical logic (factor (i)) and is able to define a function $\ulcorner \cdot \urcorner$ that assigns names to sentences (factor (ii)). Let now suppose that *T* encodes some form of naïveté (factor (iii)); more specifically, let us assume that *T* contains all the instances of the following schema:

$$ (\hbox {T-SCHEMA}) \qquad  {\mathsf {True}}(\ulcorner \varphi \urcorner ) \leftrightarrow \varphi , $$

or that *T* is closed under an *inter-substitutivity* rule, by which

$$ (\hbox {INTER-SUBSTITUTIVITY})  \qquad \varphi \in T \text{ if } \text{ and } \text{ only } \text{ if } \varphi ^{\mathsf {t}} \in T,  $$

where $\varphi ^{\mathsf {t}}$ is the result of substituting a subformula $\psi$ of $\varphi$ with ${\mathsf {True}}(\ulcorner \psi \urcorner )$ or *vice versa*. Finally, suppose that *T* can prove the existence of a sentence $\lambda$ that is equivalent to $\lnot {\mathsf {True}}(\ulcorner \lambda \urcorner )$ in *T*—the existence of sentences such as $\lambda$ can be proven in any theory that interprets a *modicum* of syntax. If *T* is non-trivial, there is a classical evaluation function *v* assigning the semantic value $\mathbf {1}$ to the sentences of *T*. What, then, is the value of $\lambda$? It is easily seen that $\lambda$ cannot be classically evaluated, on pain of contradiction. Since *v* is a classical evaluation, either $v(\lambda ) = \mathbf {1}$ or $v(\lambda ) = \mathbf {0}$. If $v(\lambda ) =\mathbf {1}$, then $v(\lnot {\mathsf {True}}(\ulcorner \lambda \urcorner )) =\mathbf {1}$ (by definition of $\lambda$), but also $v(\lnot \lambda ) =\mathbf {1}$ (courtesy of naïveté), which is absurd. We conclude that $v(\lambda ) =\mathbf {0}$, and therefore $v(\lnot {\mathsf {True}}(\ulcorner \lambda \urcorner )) =\mathbf {0}$ (by definition of $\lambda$). But the latter, by naïveté, yields $v(\lnot \lambda ) =\mathbf {0}$, which is also absurd. This is the Liar paradox.^7^

There are several options to restrict classical logic in order to non-trivially admit some form of naïveté. Semantically, this corresponds to adopting non-classical evaluation functions. Classical evaluations assign to every sentence either value $\mathbf {1}$ or value $\mathbf {0}$, and no sentence is assigned the same classical value as its negation. This sits poorly with naïveté: the Liar paradox features a sentence $\lambda$ that is forced to have the same value as its negation. However, several non-classical evaluations feature three or more semantic values: they behave as classical evaluations on classical values, but $\lambda$ and $\lnot \lambda$ can be assigned the same non-classical value. In this way, non-classical evaluations can assign the same value to $\varphi$ and ${\mathsf {True}}(\ulcorner \varphi \urcorner )$ (and thus validate inter-substitutivity or even the t-schema).^8^

In order to make the following discussion more precise, I will now introduce a family of non-classical logics that can be used to formulate several theories of naïve truth. Moreover, I will recall a few basic facts about one specific theory of naïve truth, the one developed in Field (2003, 2007, 2008), which will make it easier to discuss Field’s anti-revenge strategy. However, both Field’s anti-revenge strategy and my arguments against it are completely independent from the revenge-breeding notions and the specific theories being considered.

Let a *partial evaluation* be any function that assigns to the sentence of ${\mathcal {L}}_\mathsf{Tr}$ one of the values $\mathbf {1}$, $\mathbf {0}$, and **1/2**, and that satisfies the following criteria:

- The value of $\lnot \varphi$ is $\mathbf {1}$*minus* the value of $\varphi$.
- The value of $\varphi \wedge \psi$ is the *minimum* of the values of $\varphi$ and $\psi$.
- The value of $\forall x \varphi$ is the *infimum* of the values of its instances $\varphi (t)$.

The other logical constants are defined as usual (and evaluated accordingly): $\varphi \vee \psi$ is $\lnot (\lnot \varphi \wedge \lnot \psi )$, $\varphi \rightarrow \psi$ is $\lnot (\varphi \wedge \lnot \psi )$, and $\exists x \varphi$ is $\lnot \forall x \lnot \varphi$. Several non-classical logics that support some form of naïveté are based on partial evaluations. Strong Kleene logic, or ${\mathsf {K3}}$, is a case in point: a sentence $\varphi$ is a ${\mathsf {K3}}$-consequence of a set of sentences $\Gamma$ if, for every partial evaluation *p*, if $p(\Gamma ) = \mathbf {1}$, then $p(\varphi ) = \mathbf {1}$ (where $p(\Gamma ) = \mathbf {1}$ is a shorthand for $p(\psi ) = \mathbf {1}$, for every $\psi$ in $\Gamma$). Using ${\mathsf {K3}}$, one can give non-trivial theories of truth that validate inter-substitutivity while avoiding the truth-theoretical paradoxes.^9^

${\mathsf {K3}}$ is a very weak logic: on the one hand, several classically valid inference rules turn out to be invalid in it (notably, the classical rules for introducing negation and the conditional); on the other, ${\mathsf {K3}}$ does not have any logical laws: not even the principle $\varphi \rightarrow \varphi$ is ${\mathsf {K3}}$-valid.^10^ Several theories have been developed that strengthen ${\mathsf {K3}}$ without losing inter-substitutivity. In particular, a series of theories developed in recent years by Field (2002, 2003, 2008, 2013) succeeded in equipping ${\mathsf {K3}}$-based theories of truth with primitive, strong conditional connectives, not equivalent to ${\mathsf {K3}}$’s conditional, that validate several classically valid principles.

Field’s (2003, 2007, 2008) theory, call it ${\mathsf {F}}$, is a case in point: it extends ${\mathsf {K3}}$ with a primitive, strong conditional $\rightarrow _\mathsf{F}$, it contains all the instances of several classically valid schemata, such as $\varphi \rightarrow _{\mathsf {F}} \varphi$, $(\varphi \wedge \psi ) \rightarrow _{\mathsf {F}} \varphi$, $\varphi \rightarrow _{\mathsf {F}} (\varphi \vee \psi )$, and it satisfies inter-substitutivity and the t-schema, where the latter is formulated with Field’s biconditional, i.e. ${\mathsf {True}}(\ulcorner \varphi \urcorner ) \leftrightarrow _{\mathsf {F}} \varphi$.^11^ The set ${\mathsf {F}}$ is defined model-theoretically, namely via some evaluation function $v_{\mathscr {F}}$ from the sentences of the language to a set of semantic values (containing the classical values $\mathbf {1}$ and $\mathbf {0}$):

$$ {\mathsf {F}} = \{\varphi \in {\mathsf {SENT}} \,|\, v_{\mathscr {F}}(\varphi ) = \mathbf {1}\}, $$

where ${\mathsf {SENT}}$ indicates the set of sentences of the language of Field’s theory. The evaluation functions employed by Field to define ${\mathsf {F}}$ assign a non-classical value to sentences such as $\lambda$, thus making it possible to non-trivially retain both inter-substitutivity and the t-schema. However, ${\mathsf {F}}$ is a very expressive theory, and it possesses the resources to characterize a *determinateness operator*${\mathsf {Det}}$ that captures the status of $\lambda$ and similar sentences.^12^ Field’s determinateness operator obeys the following rules (see Field 2007, pp. 110)^13^:

$$\begin{array}{ll} \text{From } \varphi \rightarrow _\mathsf{F} \psi , \text{ infer } {\mathsf {Det}}({\varphi }) \rightarrow _\mathsf{F} {\mathsf {Det}}({\psi }) &\quad (\hbox {D1})\\ \text{From } \varphi , \text{ infer } {\mathsf {Det}}(\varphi ) &\quad (\hbox {D2}) \\ {\mathsf {Det}}({\varphi }) \rightarrow _\mathsf{F} \varphi &\quad (\hbox {D3}) \\ \text{From } \varphi \rightarrow _\mathsf{F} \lnot \varphi , \text{ infer } \lnot {\mathsf {Det}}({\varphi }) &\quad (\hbox {D4}) \end{array}$$

Thanks to its determinateness operator, Field’s theory can declare Liar sentences such as $\lambda$ as ‘not determinately true’ – indeed, the sentence $\lnot {\mathsf {Det}}({\mathsf {True}}(\ulcorner {\lambda }\urcorner ))$ is in ${\mathsf {F}}$. Field’s determinateness operator clearly allows one to form Liar-like sentences employing ${\mathsf {Det}}$ itself. The sentence $\lambda ^{*}$ inter-substitutable with $\lnot {\mathsf {True}}(\ulcorner {\mathsf {Det}}({\lambda ^{*}})\urcorner )$ in ${\mathsf {F}}$ is a case in point. Of course, the ‘indeterminate’ status of $\lambda ^{*}$ cannot be captured by declaring it ‘not determinately true’, since $\lnot {\mathsf {True}}(\ulcorner {\mathsf {Det}}({\lambda ^{*}})\urcorner )$ is inter-substitutable with $\lambda ^{*}$ itself. However, Field’s theory declares $\lambda ^{*}$ to be ‘not determinately determinately true’, and in fact $\lnot {\mathsf {Det}}({\mathsf {Det}}({{\mathsf {True}}({\urcorner \lambda\urcorner ^{*}})}) )$ is in ${\mathsf {F}}$. The iterations of ${\mathsf {Det}}$ definable in ${\mathsf {F}}$ go further, extending well into the transfinite (Field 2008, §§21–23).

${\mathsf {F}}$ provides a solution to the Liar and all the other paradoxes that can be formulated in its language: not only does it validate inter-substitutivity and a form of the t-schema, it also provides a treatment of intuitively paradoxical sentences such as $\lambda$ via (iterations of) the determinateness operator. Does this show that ${\mathsf {F}}$, and similarly expressive theories of naïve truth more generally, solve *all* the semantic paradoxes? Revenge arguments aim to answer negatively to this question. More precisely, MT-revenge arguments aim at showing that theories of naïve truth, despite solving the truth-theoretic paradoxes, fall short of solving other semantic paradoxes closely related to the ‘standard’ ones, involving other semantic notions closely related to naïve truth, definable in their MT-semantics. The upshot of revenge arguments is clear: revenge-prone theories suffer from crucial *expressive limitations*: they avoid triviality only because they cannot express the semantic notions that could trivialize them, just like classical theories cannot express naïve truth.

Here is a classic MT-revenge paradox (see e.g. Ketland 2003; Leitgeb 2007)—I present it for an unspecified theory *T*, but one could easily run it for ${\mathsf {F}}$:

**Bivalent Determinateness** Let *T* be a theory of truth that validates inter-substitutivity (but a similar argument applies for the t-schema), and let *v* be a non-classical evaluation for *T*. *T* cannot contain any operator ${\mathsf {BDet}}$ with the following semantics:

$$\begin{aligned} v({\mathsf {BDet}}(\varphi )) = {\left\{ \begin{array}{ll} \mathbf {1},&{} \text{ if } v(\varphi ) = \mathbf {1},\\ \mathbf {0},&{} \text{ if } v(\varphi ) \ne \mathbf {1} \end{array}\right. } \end{aligned}$$

To see this, let $\lambda _{\mathsf {d}}$ be inter-substitutable for $\lnot {\mathsf {True}}(\ulcorner {\mathsf {BDet}}(\lambda _{\mathsf {d}})\urcorner )$ in *T*, and apply *v* to $\lambda _{\mathsf {d}}$:

- Suppose $v(\lambda _{\mathsf {d}}) = \mathbf {1}$. By definition of ${\mathsf {BDet}}$ and naïveté, $v({\mathsf {BDet}}(\lambda _{\mathsf {d}})) = \mathbf {1} = v({\mathsf {True}}(\ulcorner {\mathsf {BDet}}(\lambda _{\mathsf {d}})\urcorner ))$. No evaluation assigns the same *classical* value to a sentence and its negation, but $\lambda _{\mathsf {d}}$ is by definition inter-substitutable for $\lnot {\mathsf {True}}(\ulcorner {\mathsf {BDet}}(\lambda _{\mathsf {d}})\urcorner )$, and therefore $v(\lnot {\mathsf {True}}(\ulcorner {\mathsf {BDet}}(\lambda _{\mathsf {d}})\urcorner )) \ne \mathbf {1} \ne v(\lambda _{\mathsf {d}})$, against our supposition.
- Suppose $v(\lambda _{\mathsf {d}}) \ne \mathbf {1}$. By definition of ${\mathsf {BDet}}$ and naïveté, $v({\mathsf {BDet}}(\lambda _{\mathsf {d}})) = \mathbf {0} = v({\mathsf {True}}(\ulcorner {\mathsf {BDet}}(\lambda _{\mathsf {d}})\urcorner ))$. Non-classical evaluations are classical on classical values, and $\lambda _{\mathsf {d}}$ is inter-substitutable for $\lnot {\mathsf {True}}(\ulcorner {\mathsf {BDet}}(\lambda _{\mathsf {d}})\urcorner )$, therefore $v(\lnot {\mathsf {True}}(\ulcorner {\mathsf {BDet}}(\lambda _{\mathsf {d}})\urcorner )) = \mathbf {1} = v(\lambda _{\mathsf {d}})$, against our supposition.

Whether $\lambda _{\mathsf {d}}$ is assigned $\mathbf {1}$ or a different value, $\lambda _{\mathsf {d}}$ and $\lnot {\mathsf {True}}(\ulcorner {\mathsf {BDet}}(\lambda _{\mathsf {d}})\urcorner )$ are impossibly forced to have the same semantic value, since ${\mathsf {BDet}}$ works as a ‘classicalizer’ for non-classical evaluations. Since *T* and *v* are arbitrary, no theory of naïve truth can feature an operator for bivalent determinateness.

Non-classical theorists typically deny the legitimacy of the above paradox, and relevantly similar ones, arguing that revenge-breeding notions such as bivalent determinateness are not genuine semantic notions.^14^ In the next section, I will discuss an anti-MT-revenge strategy elaborated by Hartry Field in a series of works (2003, 2007, 2008). Even though Field’s strategy is articulated in the context of his theory ${\mathsf {F}}$, it can be applied to every theory for which an MT-semantics can be given.^15^

## Field on MT-revenge

Field views MT-revenge arguments as the result of a confusion between *model-relative* and *model-independent* notions. Model-relative notions are essentially defined via reference to some model or evaluation function: ‘having semantic value *x*’ is a case in point. By contrast, truth is model-independent: it is characterized by principles such as the t-schema or inter-substitutivity that involve no model-theoretic reference. Field argues that genuine semantic notions are model-independent, and insofar as MT-revenge paradoxes resort to model-relative notions, they are not genuine paradoxes.

Field brings the following case in support of his claim. Consider the language of set theory expanded with a predicate ${\mathsf {True}}$ for ‘$\ldots$ is true’. A ‘highly natural’ model for this language is ‘the [classical] homophonic model whose domain consists of all non-sets together with all sets of rank less than the first inaccessible cardinal’.^16^ Call this model ${\mathscr {M}}_1$. In order to build ${\mathscr {M}}_1$, the existence of at least one inaccessible cardinal is required.^17^ However, this is where model-independent truth and its model-relative counterpart diverge:

But now consider the sentence ‘There are inaccessible cardinals’: it’s true, but false in ${\mathscr {M}}_1$, i.e. has semantic value $\mathbf {0}$ in ${\mathscr {M}}_1$; its negation is false, but has value $\mathbf {1}$ in ${\mathscr {M}}_1$. Having semantic value $\mathbf {1}$ in ${\mathscr {M}}_1$ doesn’t correspond to truth, or to determinate truth, or anything like that [$\ldots$]. The point made here for ${\mathscr {M}}_1$ applies to any other model that can be defined within set theory, by Tarski’s Theorem, and this includes all models of set theory that are at all ‘natural’. (Field 2007, p. 104)

Since model-relative notions are relativized to some models, they always capture incorrectly the *extension* of model-independent notions. Models have sets as domains. As a consequence, a model-relative semantic notion ${\mathsf {N}}$, defined relatively to a model ${\mathscr {M}}$ whose domain is *M*, can only have a subset of *M* as its extension. By contrast, model-independent notions are not relative to a particular model, and therefore their extension is not restricted to any particular set. This is exactly the divergence in extension between model-relative and model-independent truth that Field’s quote points to. And it is because of such a divergence, Field argues, that the understanding of model-independent notions is not mediated nor conveyed by MT-definitions: one cannot ‘extrapolate an understanding of a model-independent notion like truth or determinate truth from the model-relative notions’ (Field 2007, p.105). But genuine semantic notions are not model-theoretically restricted, and are therefore model-independent, or so Field’s argument suggests.

Field’s argument purports to show that model-relative semantic notions cannot be genuine semantic notions: since they are always restricted to a set, they cannot correctly capture the extension of the notion (truth, determinateness, or else) they intend to characterize. Yet, Field’s argument needs to apply even beyond model-relative notions: it needs to apply to model-independent semantic notions that are *motivated* or *justified* by the MT-semantics. As Field himself points out, revenge-breeding notions can in fact be characterized model-independently. Bivalent determinateness itself is a case in point: it is possible to (partially) *axiomatize*${\mathsf {Det}}$ in such a way that it has to obey the evaluation clauses employed in the revenge paradox of Bivalent Determinateness. I will articulate this point in the context of Field’s theory. Recall that in Field’s theory ${\mathsf {F}}$ it is possible to define a determinateness operator ${\mathsf {Det}}$ that *approximates* bivalent determinateness, in that it obeys the clauses D1–D4 introduced on page 5. Crucially, Field’s operator ${\mathsf {Det}}$ does not obey the Law of Excluded Middle:

$$ {\mathsf {Det}}(\ulcorner \varphi \urcorner ) \vee \lnot {\mathsf {Det}}(\ulcorner \varphi \urcorner ) \qquad (\hbox {D5}) $$

Adding D5 to D1–D4 would force the operator ${\mathsf {Det}}$ to behave just like the revenge-paradoxical operator ${\mathsf {BDet}}$, namely it would turn Field’s determinateness into a (partial) axiomatization of bivalent determinateness, thus trivializing the resulting theory.

Field is obviously aware of the fact that MT-revenge notions can be given model-independent formulations. Nonetheless, he argues that the resulting notions are not sufficiently well-motivated, and fail to yield genuine paradoxes.

The proponent of the [MT-]revenge problem doesn’t intend [bivalent determinateness] to be understood as model-relative. The question then arises, how is it to be understood. I do not deny that it is possible to introduce into the language an operator [$\ldots$] with many of the features that the proponent of revenge wants [i.e. D1–D4], and which is *not* model-relative. [$\ldots$] But such [operators] only breed paradox if they satisfy all the assumptions used in the [revenge-paradoxical] derivations [$\ldots$]; the one place they fail is that excluded middle [i.e. D5] cannot be assumed for them. So there is a revenge problem [$\ldots$] only if there is reason to think that we can understand a notion of [bivalent determinateness] that obeys those other assumptions *plus excluded middle*.

And why assume that? I think what underlies the [MT-]revenge problem is the thought that the model-relative [bivalent determinateness operators] all obey excluded middle, so there must be an absolute [operator] that does too. But this assumption seems to me completely unwarranted: one just can’t assume that one can extrapolate in this way from the case of model-relative predicates, which make sense only by virtue of ‘misinterpreting’ the quantifiers as having restricted range, to the unrelativized case where no such ‘misinterpretation’ is in force. (Field 2007, pp. 108–109)

The model-independent notion of bivalent determinateness (obeying D1–D5), Field argues, is only motivated by his model-relative counterparts: but how can the latter justify the former, given that model-relative notions fall inevitably short of determining the extension of the model-independent ones?

Summing up, Field’s argument is based on the misalignment between model-independent and model-relative notions: the latter always fail to capture the extension of the former. However, model-relative notions can be given model-independent (revenge-breeding) formulations. Therefore, if Field’s strategy is to succeed, his argument must apply to model-independent notions as well. For this reason, Field’s argument needs some principle that links a sufficient understanding of a model-independent notion with its extension. Here’s a first stab at such a bridge principle:

(extension): A sufficiently clear understanding of a model-independent notion ${\mathsf {N}}$ entails the existence of a criterion to determine the extension of ${\mathsf {N}}$.

The need for a criterion to determine ${\mathsf {N}}$’s extension is suggested by Field’s argument itself: one can be agnostic about the existence of inaccessible cardinals, but a sufficiently clear understanding of naïve truth requires the sentence ‘There are inaccessible cardinals’ to be in the extension of ${\mathsf {True}}$ just if there are large cardinals. Understanding truth, therefore, requires a suitable criterion to determine the extension of the corresponding notion.^18^ If one wants to endorse Field’s anti-revenge strategy, she has to endorse something like extension. Not accepting extension would amount to not accepting the only ground to discriminate between naïve truth and model-relative notions that Field’s argument offers, namely that model-relative notions lack an extension which is not incorrectly determined.

Field’s argument does not rely on the specificities of his theory, or of bivalent determinateness: if successful, it would defuse *every* MT-revenge argument. However, in the next section I’ll argue that Field’s argument must ultimately be rejected: MT-semantic notions, with their potential for revenge, still stand.^19^

## Intelligibility and extensions

extension effectively extends Field’s argument to model-independent notions motivated by the MT-semantics. However, it is too weak. The schemata D1–D5 that characterize bivalent determinateness model-independently provide criteria to determine its extension just like the t-schema or inter-substitutivity does for truth. Therefore, extension does not suffice to conclude that naïve truth is a genuine semantic notion, while D1-D5-determinateness is not, which is what Field’s strategy aims to accomplish.

More precisely, the t-schema provides a criterion to determine the extension of ${\mathsf {True}}$, according to which:

- ‘$2+2 = 4$’ is in the extension of ${\mathsf {True}}$ if and only if $2+2 = 4$,
- ‘there are inaccessible cardinals’ is in the extension of ${\mathsf {True}}$ if and only if there are inaccessible cardinals,

But D1–D5 can also be read as criteria to determine, perhaps partially but at least not incorrectly, the extension of ${\mathsf {Det}}$:^20^

D1: - From a derivation of ‘if $2+2 = 4$ then $3+2 = 5$’, infer that if ‘$2+2 = 4$’ is in the extension of ${\mathsf {Det}}$ then ‘$3+2 = 5$’ is in the extension of ${\mathsf {Det}}$,
  - From a derivation of ‘if there are inaccessible cardinals then there are large cardinals’, infer that if ‘there are inaccessible cardinals’ is in the extension of ${\mathsf {Det}}$ then ‘there are large cardinals’ is in the extension of ${\mathsf {Det}}$,
D2: - From a derivation of ‘$2+2 = 4$’, infer that ‘$2+2 = 4$’ is in the extension of ${\mathsf {Det}}$,
  - From a derivation of ‘there are inaccessible cardinals’, infer that ‘there are inaccessible cardinals’ is in the extension of ${\mathsf {Det}}$,
D3: - if ‘$2+2 = 4$’ is in the extension of ${\mathsf {Det}}$, then $2+2 = 4$,
  - if ‘there are inaccessible cardinals’ is in the extension of ${\mathsf {Det}}$, then there are inaccessible cardinals,
D4: - From a derivation of ‘if $2+2 = 4$ then it is not the case that $2+2 = 4$’, infer that ‘$2+2 = 4$’ is in the extension of the negated ${\mathsf {Det}}$,
  - From a derivation of ‘if there are inaccessible cardinals then there are not inaccessible cardinals’, infer that ‘there are inaccessible cardinals’ is in the extension of the negated ${\mathsf {Det}}$,
D5: - Either ‘$2+2 = 4$’ is in the extension of ${\mathsf {Det}}$ or ‘$2+2 = 4$’ is in the extension of the negated ${\mathsf {Det}}$,
  - Either ‘there are inaccessible cardinals’ is in the extension of ${\mathsf {Det}}$ or ‘there are inaccessible cardinals’ is in the extension of the negated ${\mathsf {Det}}$,

Summing up, extension is too weak to salvage Field’s anti-revenge strategy because model-independent bivalent determinateness, i.e. ${\mathsf {Det}}$ axiomatized by D1–D5, *also* satisfies extension. D1–D5 provide criteria to determine the extension of bivalent determinateness in Field’s theory—even though they make it trivial—and therefore extension fails to exclude bivalent determinateness from the genuine semantic notions.

In order to apply Field’s anti-revenge strategy, and deny that D1–D5-bivalent determinateness is a genuine semantic notion, one has to exclude trivial concepts from the application of extension. This is the only option to salvage Field’s argument, because naïve truth and bivalent determinateness (a) are both formulated in purely model-independent terms, and (b) both enjoy criteria to determine their extension (not incorrectly). For this reason, Field’s argument really needs to be supplemented with the following principle:

(c-extension): A sufficiently clear understanding of a model-independent notion ${\mathsf {N}}$ entails the existence of a criterion that determines the extension of ${\mathsf {N}}$, and it requires ${\mathsf {N}}$’s extension to be non-trivial.

c-extension is a very strong principle: it entails that if a notion ${\mathsf {N}}$ breeds paradox for a theory *T*, than ${\mathsf {N}}$ is not understandable for anyone accepting *T*. This seems deeply problematic, for it turns any attempt to compare theories into a futile exercise: the advocates of *T* would declare every notion incompatible with *T* to be simply nonsense. Several fundamental debates in theories of truth—including the debates on which is the ‘right’ non-classical logic of naïve truth, and the debates on whether truth is naïve—would also have to be considered completely pointless. Yet, this is what theorists that aim to avoid revenge via Field’s argument ought to believe: as the foregoing discussion shows, they ought to believe that notions whose extension can be determined with a precise criterion *but trivializeT* are just not understandable enough.^21^ I now highlight the consequences of c-extension with a concrete, and well-known, example:

**Incompatible negations** Let us consider again the logic ${\mathsf {K3}}$ (see page 4). ${\mathsf {K3}}$-logical validity is defined as preservation of value $\mathbf {1}$ in every partial evaluation. However, other logics can be defined from partial evaluations that are weak enough to support forms of naïveté: the *logic of paradox*, ${\mathsf {LP}}$, is a case in point.^22^ A sentence $\varphi$ is a ${\mathsf {LP}}$-consequence of a set of sentences $\Gamma$ if, for every partial evaluation *p*, if $p(\Gamma ) = \mathbf {1}$ or **1/2**, then $p(\varphi ) = \mathbf {1}$ or **1/2**. ${\mathsf {K3}}$- and ${\mathsf {LP}}$-based theories are dual in several respects. In particular, ${\mathsf {K3}}$-based theories do not satisfy all instances of the law of excluded middle (${\mathsf {LEM}}$) $\varphi \vee \lnot \varphi$, but satisfy all instances of *ex falso quodlibet* (${\mathsf {EFQ}}$) $\varphi \wedge \lnot \varphi \vdash \psi$, while ${\mathsf {LP}}$-based theories validate all the instances of ${\mathsf {LEM}}$, but fail to validate all the instances of ${\mathsf {EFQ}}$. Since the ${\mathsf {K3}}$-negation trivialize ${\mathsf {LP}}$-based theories (and *vice versa*), accepting c-extension would force advocates of ${\mathsf {K3}}$-based theories to declare the ${\mathsf {LP}}$-negation unintelligible (and *vice versa*), even though both excluded middle and *ex falso quodlibet* are classically valid and customarily employed in (formalized) mathematical reasoning.

Can c-extension be defended even after one realizes its implications for the comparisons of theories of truth? The foregoing considerations should give the advocate of c-extension pause. However, there are far more conclusive reasons to reject this principle: in fact, c-extension seems to clash directly with the intelligibility of naïve truth. If c-extension is correct, it applies across the board, and not just to revenge-breeding semantic notions. Its application to naïve truth shows that this notion was not sufficiently understandable before the development of suitable non-classical theories in relatively recent times. At the very least, c-extension entails that naïve truth was not sufficiently understandable when it was first formulated as a limitative result (see e.g. Tarski 1936) in that it yields triviality once added to the accepted formal systems of first-order arithmetic (or some other sufficiently expressive theory) formulated in classical logic.

One might try to save c-extension postulating two senses of ‘understanding’. In a ‘shallow’ sense, one understands semantic notions irrespectively of their extensions and of whether they trivialize other notions. In a ‘deep’ sense, understanding semantic notions requires c-extension to be satisfied. Some form of shallow/deep distinction is customary in scientific domains: non-specialists understand shallowly notions such as *computable*, *black hole*, or *DNA*, while specialists understand these notions properly. Understanding semantic notions properly might require to consult the relevant experts, while the shallow sense ‘saves the appearances’ according to which everyone can understand naïve truth and bivalent determinateness. However, in order to use c-extension to escape revenge paradoxes, one must suppose that only the deep sense is relevant to determine which semantic notions and paradoxes there are. But there are at least two problems with this supposition. First, the community of truth-theorists is split over which theory of truth is correct. Therefore, one accepting c-extension has to conclude that there are experts lacking a deep understanding of genuine semantic notions *just because they disagree* (for discussion, see Williamson 2007, Chapter 4). Second, this supposition is problematic for the reasons highlighted above. Before non-classical theories were available, naïve truth was only shallowly understandable but it generated genuine paradoxes, such as the Liar paradox. Why, then, should bivalent determinateness fail to generate genuine paradoxes?

One could further object that the understanding of naïve truth is and has always been (at least implicitly) coupled with some restriction of classical logic that avoids triviality. From this perspective, the understanding of naïve truth is and has always been perfectly in line with c-extension. Now, this objection might in effect provide a construal of c-extension that does not make it immediately implausible, although independent support would be needed to show that the understanding of naïve truth is indissolubly coupled with a suitable non-classical logic. However, this objection fails to vindicate Field’s anti-revenge argument, for it is equally applicable to bivalent determinateness. If naïve truth trivializes classical theories but it is to be understood as an essentially non-classical concept, then the same could be said for bivalent determinateness: this notion trivializes several currently available non-classical theories but it is also to be understood as a (super-)non-classical concept that requires extremely weak non-classical theories. Therefore, this objection does not separate naïve truth from bivalent determinateness: it shows that, in both cases, a non-classical logic that makes these notions non-trivial is required to claim an understanding of them. But this does not make bivalent determinateness any less acceptable than naïve truth.^23^

In conclusion, Field’s argument does not offer an acceptable way to distinguish between naïve truth and bivalent determinateness so that the former can be claimed to generate genuine paradoxes while the latter can’t. Since nothing in the foregoing arguments relies on the specificities of bivalent determinateness or Field’s theory, I conclude that Field’s argument does not offer a way out of revenge paradoxes motivated by the MT-semantics. In the next section, I consider a possible criterion to rule out MT-semantic notions, that implicitly informs several anti-revenge strategies, and argue that it also fails to provide a convincing base to distinguish ‘revenge-breeding’ from ‘standard’ paradoxical notions.

## Model-theoretic instrumentalism

As Field’s argument makes clear, naïve truth and D1–D5-bivalent determinateness are separated by their conceptual justification: the latter is motivated by the MT-semantics of an object-theory, while the former isn’t. If one could show that the MT-semantics cannot motivate genuine semantic notions, one could argue that bivalent determinateness does not yield a genuine paradox. Some authors view the MT-semantics as a mere *instrument* to prove the object-theory non-trivial; as such, the MT-semantics might not allow one to carve out genuine semantic notions (see e.g. Beall (2007, pp. 10–11) and Yablo (2003, pp. 328–329)). Call this position MT-instrumentalism. One possible motivation for it is that different MT-semantics can be given in different meta-theories. Some MT-semantic notions definable in a classical meta-theory might not be definable in a *non-classical* meta-theory, including revenge-breeding notions such as the one employed in the paradox of Bivalent Determinateness.

Nevertheless, MT-instrumentalism does not provide a way out of the MT-revenge paradoxes. In short, even if the MT-semantics is regarded as a mere tool to prove the non-triviality of a theory *T*, there are crucial facts about the logical behaviour of *T*’s sentences that can be captured in *any* MT-semantics for *T*. More precisely, *every* MT-semantics for *T* can distinguish sentences that respect all the principles of classical logic from sentences that obey distinctively non-classical principles. This shows that MT-semantics track genuine semantic distinctions, irrespectively of the specific models being employed, and thus irrespectively of the restrictions imposed on the extension of semantic notions from the set-theoretic nature of models.

I will again articulate this point in connection with Field’s theory, although the argument generalizes easily. Field’s theory ${\mathsf {F}}$ (sketched on page 4) is an infinite set of sentences that contains all the instances of several classical laws and of the t-schema (formulated with Field’s conditional $\rightarrow _{\mathsf {F}}$), and it is closed under several classical rules of inference and inter-substitutivity. Recall, the set ${\mathsf {F}}$ is defined model-theoretically, namely via some evaluation function $v_{\mathscr {F}}$ from the sentences of the language to a set of semantic values. ${\mathsf {F}}$ consists of the sentences to which $v_{\mathscr {F}}$ assigns the value $\mathbf {1}$:

$$ {\mathsf {F}} = \{\varphi \in {\mathsf {SENT}} \,|\, v_{\mathscr {F}}(\varphi ) = \mathbf {1}\} $$

where ${\mathsf {SENT}}$ indicates the set of sentences of the language of Field’s theory. Let’s say that an evaluation function $v_{\mathscr {F}}$ from ${\mathsf {SENT}}$ to a set of semantic values *defines*${\mathsf {F}}$ if ${\mathsf {F}} = \{\varphi \in {\mathsf {SENT}} \,|\, v_{\mathscr {F}}(\varphi ) = \mathbf {1}\}$. For the MT-instrumentalist, *how*${\mathsf {F}}$ is defined is completely insubstantial. In particular, ${\mathsf {F}}$ could be defined by an evaluation $v^{n}_{\mathscr {F}}$ defined in a non-classical meta-theory, in which it is not always the case that $v^{n}_{\mathscr {F}}(\varphi ) = \mathbf {1}$ or $v^{n}_{\mathscr {F}}(\varphi ) \ne \mathbf {1}$.

Now, ${\mathsf {F}}$ is a non-classical theory, and therefore the principles of classical logic hold for some of ${\mathsf {F}}$’s sentences, while other sentences exhibit a non-classical behaviour. Suppose $v^*_{\mathscr {F}}$ is an evaluation that defines ${\mathsf {F}}$. In $v^*_{\mathscr {F}}$, we have

$$ v^*_{\mathscr {F}}\big (\lnot [\forall x (x=x) \leftrightarrow _{\mathsf {F}} \lnot \forall x (x=x)]\big ) = \mathbf {1} \quad \text { and } \quad v^*_{\mathscr {F}}\big (\lambda \leftrightarrow _{\mathsf {F}} \lnot \lambda \big ) = \mathbf {1}. $$

The biconditional of Field’s logic $$\leftrightarrow _{\mathsf {F}}$$ is non-classical, so one should expect it to behave non-classically. Still, we know that $$(\forall x (x=x) \vee \lnot \forall x (x=x))$$ is in $${\mathsf {F}}$$, and that if $$\varphi \vee \lnot \varphi $$ is in $${\mathsf {F}}$$, then $$\varphi $$ is closed under all the classical principles involving $$\varphi $$, and also the classical principles for the (otherwise non-classical) $$\leftrightarrow _{\mathsf {F}}$$. This is enough to conclude that, in any evaluation that defines $${\mathsf {F}}$$, the laws of identity and Liar sentences have different logical behaviours: while $$\forall x (x=x)$$ obeys all the classical principles (including $$\lnot (\varphi \leftrightarrow _{\mathsf {F}} \lnot \varphi )$$) because it obeys the enabling condition by which $$\leftrightarrow _{\mathsf {F}}$$ behaves just like the classical biconditional on it, $$\lambda $$ obeys the non-classical principle $$\varphi \leftrightarrow _{\mathsf {F}} \lnot \varphi $$.^24^$$^{,}$$^25^

Another way to discern the non-classical logical behaviour of some sentences in ${\mathsf {F}}$ uses Field’s very notion of D1-D4-determinateness: in *any* evaluation function $v^*_{\mathscr {F}}$ that defines ${\mathsf {F}}$, $v^*_{\mathscr {F}}\big ({\mathsf {Det}}(\forall x (x=x))\big ) = \mathbf {1}$ and $v^*_{\mathscr {F}}\big (\lnot {\mathsf {Det}}(\lambda )) = \mathbf {1}$. A moment’s reflection shows that some grasp of the non-classicality of sentences such as $\lambda$ is at least presupposed for Field’s theory. For, it if weren’t, what could have motivated a determinateness operator that describe its ‘indeterminateness’ in the first place?^26^

The foregoing observations show that any MT-semantics tracks crucial object-theoretic facts. This does not depend on whether one attributes an instrumental role to the MT-semantics, and it is also independent on the specific evaluation functions employed, and thus on the specific models on which they are defined. Therefore, even the MT-instrumentalist has to concede that the MT-semantics, whether developed in a classical or a non-classical meta-theory, can be used to articulate genuine semantic distinctions. This lesson finds a natural application to revenge paradoxes: they make explicit, via paradoxical notions, some semantic distinctions that can be made, if only partially, in the MT-semantics. For instance, bivalent determinateness makes explicit the different logical behaviour of sentences such as $\lambda$ and $\forall x (x=x)$.

An advocate of Field’s theory (or of another sufficiently expressive theory) might object at this point that the different logical behaviours of $\lambda$ and $\forall x (x=x)$ can already be captured by a determinateness operator obeying D1-D4 alone, since both ${\mathsf {Det}}(\forall x (x=x))$ and $\lnot {\mathsf {Det}}({\lambda })$ are in ${\mathsf {F}}$. If one then tries to formulate revenge-paradoxical sentences, the objection continues, their non-classical behaviour can be captured iterating D1-D4-determinateness. Recall the sentence $\lambda ^{*}$ equivalent to $\lnot {\mathsf {True}}(\ulcorner {\mathsf {Det}}(\lambda ^{*})\urcorner$, mentioned on page 5: its non-classical behaviour cannot be captured by declaring it ‘not determinately true’, since it is equivalent to $\lambda ^{*}$. However, Field’s theory declares $\lambda ^{*}$ to be ‘not determinately determinately true’, since $\lnot {\mathsf {Det}}({\mathsf {Det}}({{\mathsf {True}}({\urcorner\lambda \urcorner ^{*}})}) )$ is in ${\mathsf {F}}$. And so on. However, Welch (2014) has shown that if the iteration is continued long enough, ‘ineffable Liars’ can be defined in ${\mathsf {F}}$, namely sentences whose non-classical behaviour cannot be captured by *any* iteration of determinateness that can be constructed in ${\mathsf {F}}$ (see also Rayo and Welch 2007). Yet, the non-classical behaviour of Welch’s ineffable Liars can be characterized very easily in several MT-semantics for ${\mathsf {F}}$, simply using the notion of bivalent determinateness.

In conclusion, the MT-semantics might be thought to have primarily an instrumental role, but it also provides crucial information about the behaviour of sentences of the object-theory. For this reason, the MT-semantics can be used to carve out genuine semantic notions, that make such behaviour explicit. These notions may be paradoxical, but cannot be thought of as the by-product of a mere instrument.^27^

## Concluding remarks

Piet Hein famously quipped that ‘problems worthy of attack prove their worth by hitting back’. The growing attention which truth theorists devote to revenge paradoxes witnesses that they are considered to be worthy of attack. I hope to have shown that they do hit back.

In particular, Field’s anti-revenge-strategy does not offer a way out of the MT-revenge paradoxes. The relevance of Field’s strategy is hardly over-estimated, since it is applicable to every theory of truth that has an MT-semantics, and it threatens to undermine the coherence of MT-semantic notions, or notions motivated by the MT-semantics, more generally. However, Field’s defence, once fully articulated, offers no acceptable reason to think that revenge-breeding notions are not genuine semantic notions. MT-instrumentalism is equally unsuccessful as a strategy to reject revenge-breeding MT-notions, for they can be seen to carve out genuine semantic distinctions about the object-theories.

As emphasized in Sect. 1, the foregoing arguments do not show that every MT-revenge paradox is successful; they show that MT-revenge paradoxes are not to be dismissed *just because* they involve the MT-semantics. However, this modest conclusion has implications for our understanding of semantic paradoxes, and ultimately for deciding amongst approaches to treating truth and other semantic notions in the object-language. One such implication is that there is no well-motivated ground to distinguish between ‘standard’ and ‘revenge’ paradoxes. Cogently motivated revenge-breeding notions are legitimate semantic notions, just like naïve truth. Revenge paradoxes are not *ex post facto* antinomies, that only arise ‘after’ a solution to the ‘standard’ paradoxes has been found, and target that specific solution: they are expressive limitations that have been affecting the language all along, exactly as the ‘standard’ paradoxes. The whole distinction between ‘revenge’ and ‘standard’ paradoxes should be abandoned.

But if there is no such a thing as a ‘standard’ versus ‘revenge’ distinction, then a theory of truth that treats successfully the ‘standard’ paradoxes but fails to treat the ‘revenge’ ones is just a theory that solves the paradoxes only partially. For example, if naïve truth and bivalent determinateness are equally legitimate notions, the very logical revision that is operated to express the former is not fully justified if it cannot also be used to express the latter. So, abandoning the ‘standard’ versus ‘revenge’ distinction can help decide among theories of truth and treatments of paradoxes.^28^

One might worry that rehabilitating revenge paradoxes would have devastating consequences. Since revenge paradoxes are so pervasive, is there still hope for theories that express all the genuine semantic notions? Field himself seems to share such a worry:

The strong revenge worry is that adding such an operator [bivalent determinateness] to the language would produce a new paradox that requires giving up the truth schema. Substantiating this would be a fatal blow to any claim that a G-solution [the kind of solutions he favours] adequately resolves all the paradoxes. (Field 2007, p. 120)

However, there is no a priori reason to think that no theory can express all the semantic notions in the object-language. The semantic paradoxes—‘standard’ and ‘revenge’ alike—simply show that deep expressive limitations stand on the way to this goal. What cannot be reasonably hoped for is a theory of truth and other semantic notions that is completely free from expressive limitations. What can be reasonably hoped for is a theory that addresses such expressive limitations *uniformly*—thus applying the same solution to ‘standard’ and ‘revenge’ paradoxes. From this point of view, some classical theories seem promising: in particular, *contextualist theories* can be given that offer the very same solution to *all* semantic paradoxes, while retaining the strength of full classical logic.^29^

## Footnotes

1 For simplicity, I will only consider semantic notions formalized as first-order predicates and operators.

2 In other words, neither the sentence ‘$\lambda$ is true’ nor the sentence ‘$\lambda$ is not true’ are in *T*. See for example the theories developed in Kripke (1975), Field (2003, 2008) and Halbach and Horsten (2006).

3 Revenge paradoxes also exist for *classical* theories of truth (see e.g. Bacon 2015): my arguments for MT-revenge generalize to classical theories as well, but I will not explicitly discuss classical approaches, in the interest of space.

4 See e.g. Beall (2007) and Field (2007, 2008).

5 My focus in this paper is on MT-revenge and on Field’s treatment thereof. Several general treatments of revenge paradoxes can be found in the literature, see e.g. Beall (2006, 2007, 2007b), Cook (2007), Eklund (2007), Maudlin (2007), Priest (2007), Restall (2007), Scharp (2007, 2013), Simmons (2007, Shapiro 2011 and Scharp (2013). In particular, Ketland (2003) and Beall (2007) discuss MT-revenge paradoxes in connection with classical meta-theories, and Leitgeb (2007) focuses on revenge paradoxes affecting the theory developed in Field (2003, 2007, 2008) in classical and non-classical meta-theories. In this paper, I focus on Field’s argument, since it is meant to apply to every MT-semantics, irrespective of whether they are defined in a classical or non-classical meta-theory, and it applies to every theory of truth that has been given an MT-semantics.

6 Actually, semantic paradoxes arise also in sufficiently strong, but non-classical, logics, such as intuitionistic logic. I will focus on classical logic for simplicity.

7 In keeping with the model-theoretic focus of this paper, I present the Liar and other paradoxes semantically, and I only consider theories of truth for which an MT-semantics has been developed. While these restrictions rule out theories that have only been developed axiomatically (e.g. Zardini 2011), nothing prevents the arguments in this paper from applying to MT-semantics that have yet to be developed.

8 Several many-valued logics can be given two-valued semantics: see Chemla et al. (2017) for a systematic study and Rosenblatt (2015) for applications to theories of truth. However, MT-revenge paradoxes can be reproduced in such alternative two-valued frameworks, and therefore I will not explicitly consider them.

9 For more on ${\mathsf {K3}}$, see Kleene (1952) and Blamey (2002). For theories of naïve truth employing ${\mathsf {K3}}$ (or closely related logics), see Kripke (1975), Kremer (1988), Halbach and Horsten (2006) and Horsten (2009).

10 The reason for this is easily stated: while $\mathbf {1}$ is the only designated value of ${\mathsf {K3}}$, any formula whose subformulae are assigned value **1/2** by a partial evaluation *p* is itself assigned value **1/2** by *p*, so no formula receives value $\mathbf {1}$ under all partial evaluations. For example, if $p(\varphi ) =$**1/2**, then $p(\varphi \rightarrow \varphi ) =$**1/2** as well.

11 See Field (2003, §4) and Field (2008, Chapter17.4) for a partial list of the principles validated in Field’s theory. See Rossi (2016) for a self-contained presentation of Field’s theory, and a discussion of its conditional.

12 For simplicity, the notion of determinateness is treated as an operator ${\mathsf {Det}}$, namely as a syntactic connective applying to sentences. A determinateness *predicate*, applying to names of sentences, is however easily definable in ${\mathsf {F}}$, because ${\mathsf {F}}$ satisfies inter-substitutivity. So, a determinateness predicate ${\mathsf {DT}}$ obeying exactly the same principles of the operator ${\mathsf {Det}}$ can be defined putting ${\mathsf {DT}}(\ulcorner \varphi \urcorner ) := {\mathsf {True}}(\ulcorner {\mathsf {Det}}(\varphi )\urcorner )$.

13 As Field notes, D3 might be strengthened to the following condition: ‘From $\varphi \rightarrow _{\mathsf {F}} {\mathsf {Det}}(\varphi )$, infer $\varphi \vee \lnot \varphi$’. I will ignore this possibility for the sake of simplicity.

14 The legitimacy of MT-revenge paradoxes cannot be questioned on purely formal grounds: ${\mathsf {BDet}}$ is definable in minimally strong meta-theories (Leitgeb 2007). One could object that ${\mathsf {BDet}}$ is definable only because a *classical* meta-theory is employed (Yablo 2003; Leitgeb 2007); a suitable non-classical meta-theory would make it undefinable. However, too little is known about (suitable) non-classical meta-theories to tell whether they would make *all* revenge-breeding notions undefinable. In Sect. 5 I present an argument for MT-revenge that does not rely on the classicality of one’s meta-theory. For a non-classical meta-theory for a relatively weak object-theory, see Bacon (2013); for recent investigations on non-classical meta-theories, see Field et al. (2017).

15 Using bivalent determinateness as a representative revenge paradox in the context of Field’s theory may seem inappropriate, especially in view of verdicts such as the following one: ‘*there can be no truth-like predicate for which excluded middle can be assumed*’ (Field 2007, p. 89, emphasis in the original). Nevertheless, Field acknowledges that ‘the conviction that there *must* be truth-like predicates obeying excluded middle is one primary source of revenge worries’ (*ibidem*). Likewise, he concedes that an argument against his theory based on bivalent determinateness ‘is perhaps the one with most intuitive force: it is that we just need a unified [(i.e. bivalent)] account of determinacy or defectiveness’ (Field 2008, p. 140)—I thank an anonymous referee for pointing out the latter quote to me. In any case, in the context of Field’s theory, paradoxes structurally similar to the paradox of bivalent determinateness can be given without resorting to bivalence, e.g. using idempotent determinateness.

16
Field (2007, p. 104). Homophonic models ‘assign to a name its real bearer and analogously for function symbols, and […] in the classical case assign to a predicate those objects in its real extension *that are also in the domain of the model*.’ (Field 2007, *ibidem*) Field assumes for the sake of the example that there are inaccessible cardinals. If there are no inaccessible cardinals, the example can be altered accordingly.

17 This is a basic fact of Zermelo-Fraenkel Set Theory. See for example Jech (2002, pp. 167–168).

18 There are several options for such a criterion, e.g. Dummett’s criterion of applicability (Dummett 1993, pp. 75–77 and pp. 232–235). extension does not require a criterion that is effectively applicable, nor a criterion that determines completely the extension of a given notion. Field’s argument only requires the extension of a notion ${\mathsf {N}}$ to be determined *not incorrectly*: the sentence ‘there are large cardinals’ should not fall outside of the extension of ${\mathsf {True}}$ if there are large cardinals.

19 The relevance of Field’s argument goes well beyond revenge paradoxes: if it were successful, it would have a profound impact on most truth-conditional semantic theories. On the one hand, Field’s argument tells against any approach to semantics that relies heavily on model-theoretical resources, including several theories in the Montagovian tradition (see e.g. Chierchia and McCconnell-Ginet (2000); for an argument against Montagovian semantics based on their use of model-relative notions, see Lepore (1983); for a recent reply, see Glanzberg (2014)). On the other, Field’s argument tells against several theories in the tradition of Davidson (1967), which are based on model-independent notions motivated or justified by the MT-semantics (see e.g. Fischer et al. (2015, §3.1), Halbach (2014, Chapter 8)). Given its scope, one might worry that Field’s anti-revenge argument rules out every approach to truth-conditional semantics. However, the deflationary approach to truth-conditions articulated in Field (1994) might be used to answer to this worry. See also Hill (2014, Part I) and McGee (2016) for a recent discussion.

20 Standardly, predicates have extension and operators don’t. However, I’m ignoring this fact, given that in the presence of inter-substitutivity one can equally treat bivalent determinateness, or other semantic notions, as predicates or operators. See footnote 12.

21 Besides being conceptually problematic, the idea that comparing theories of truth is a futile exercise is disproven by the truth-theoretic literature. On the one hand, proponents of non-classical theories devote considerable efforts in exploring and highlighting the advantages of their approach over their rivals, classical and non-classical alike [see e.g. Beall (2009, Chapters 4–5), Field (2008, Chapters 7–8, 10–14, 24–26), Priest (2006, Chapter 20)]. On the other hand, advocates of classical theories of truth have sought to expose the limitations of non-classical approaches, from mathematical and from broadly methodological standpoints [see e.g. Halbach (2014, Chapter 20), Halbach and Nicolai (2017), Williamson (2017), Murzi and Rossi (2018a)].

22 See Asenjo (1966) and Priest (1979).

23 Alternatively, one might think that the classical logician has an understanding of naïve truth via her acceptance of a collection of instances of the naïve principles that does not yield triviality (see e.g. Horwich (1990), and McGee (1992), and Cieśliński (2007) for criticisms). However, this alternative objection would also fail to separate naïve truth from bivalent determinateness, thus failing to undermine the revenge problem posed by the latter notion.

24 In Murzi and Rossi (2018a), purely object-linguistic revenge paradoxes are developed that exploit the characterization of sentences obeying, or failing to obey, all the principles of classical logic.

25 Were the meta-theory in which $v^*_{\mathscr {F}}$ is constructed sufficiently strong to prove $v^*_{\mathscr {F}}(\lambda \vee \lnot \lambda ) \ne \mathbf {1}$, the non-classical behaviour of $\lambda$ would be more evident. However, that $v^*_{\mathscr {F}}(\lambda \vee \lnot \lambda ) \ne \mathbf {1}$ is not a fact about ${\mathsf {F}}$, but about what is *not* in ${\mathsf {F}}$, and it might be out of reach for non-classical meta-theories. Nevertheless, the facts mentioned above suffice to conclude that $\lambda$ and $\forall x(x=x)$ have different logical behaviours.

26 Accepting some form of non-classicality for $\lambda$ seems to be a prerequisite to determine the attitudes a rational agent should have towards $\lambda$ (see Caie 2012).

27 The MT-instrumentalist could object that the very idea of *defining* an object-theory via evaluation functions, i.e. MT-semantic notions, is misguided. For instance, Field (2008, p. 277) argues that the MT-semantics could be seen just as offering ‘a proof that one won’t get into trouble operating with the inferences in it’, although what is validated by the MT-semantics ‘outruns ‘real validity”. However, such a position offers no way out of the foregoing arguments for the significance of MT-notions. Even if one could select a subset ${\mathsf {F}}_0$ of ${\mathsf {F}}$ which is simple enough to be characterized axiomatically, the distinctions concerning the logical behaviour of ${\mathsf {F}}$’s sentences that can be made in the MT-semantics would apply to ${\mathsf {F}}_0$’s sentences just as well.

28 One could object that the revenge-breeding notions are somewhat less important than truth (see e.g. Maudlin 2007). Yet, the discussion in Sect. 5 makes it clear that revenge-breeding notions can express key facts of the object-theories.

29 See Glanzberg (2004), Parsons (1974), Simmons (1993, 2015) and Murzi and Rossi (2018b).
